# Supplementary material for: Learning tissue representation by identification of persistent local patterns in spatial omics data
Source: Nat Commun. 2025 Apr 30;16:4071. doi: 10.1038/s41467-025-59448-0 (PMC12044154; doi:10.1038/s41467-025-59448-0)
Supplement: Supplementary file 2 — Reporting Summary [file 41467_2025_59448_MOESM2_ESM.pdf]

nature portfolio

Corresponding author(s): Jovan Tanevski and Julio Saez-Rodriguez

Last updated by author(s): Mar 25, 2025

Reporting Summary

Nature Portfolio wishes to improve the reproducibility of the work that we publish. This form provides structure for consistency and transparency in reporting. For further information on Nature Portfolio policies, see our Editorial Policies and the Editorial Policy Checklist.

Statistics

For all statistical analyses, confirm that the following items are present in the figure legend, table legend, main text, or Methods section.

n/a	Confirmed	
☐	☒	The exact sample size (*n*) for each experimental group/condition, given as a discrete number and unit of measurement
☐	☒	A statement on whether measurements were taken from distinct samples or whether the same sample was measured repeatedly
☐	☒	The statistical test(s) used AND whether they are one- or two-sided<br>*Only common tests should be described solely by name; describe more complex techniques in the Methods section.*
☐	☒	A description of all covariates tested
☐	☒	A description of any assumptions or corrections, such as tests of normality and adjustment for multiple comparisons
☐	☒	A full description of the statistical parameters including central tendency (e.g. means) or other basic estimates (e.g. regression coefficient) AND variation (e.g. standard deviation) or associated estimates of uncertainty (e.g. confidence intervals)
☐	☒	For null hypothesis testing, the test statistic (e.g. *F*, *t*, *r*) with confidence intervals, effect sizes, degrees of freedom and *P* value noted<br>*Give P values as exact values whenever suitable.*
☒	☐	For Bayesian analysis, information on the choice of priors and Markov chain Monte Carlo settings
☒	☐	For hierarchical and complex designs, identification of the appropriate level for tests and full reporting of outcomes
☒	☐	Estimates of effect sizes (e.g. Cohen's *d*, Pearson's *r*), indicating how they were calculated

Our web collection on statistics for biologists contains articles on many of the points above.

Software and code

Policy information about availability of computer code

Data collection	No software was used for data collection
Data analysis	Kasumi is implemented as a modular open-source R package freely available from https://www.github.com/jtanevski/kasumi . The code for the implementation of Kasumi that was used to analyze the data and produce the results is freely available from https://www.github.com/saezlab/kasumi_bench. The exact version of the code together with streamlined examples of running Kasumi on the CTCL CODEX dataset and the PDAC SMI dataset and all result databases are available at https://doi.org/10.5281/zenodo.14891956 .

For manuscripts utilizing custom algorithms or software that are central to the research but not yet described in published literature, software must be made available to editors and reviewers. We strongly encourage code deposition in a community repository (e.g. GitHub). See the Nature Portfolio guidelines for submitting code & software for further information.

Data

Policy information about availability of data

All manuscripts must include a data availability statement. This statement should provide the following information, where applicable:

- Accession codes, unique identifiers, or web links for publicly available datasets
- A description of any restrictions on data availability
- For clinical datasets or third party data, please ensure that the statement adheres to our policy

The datasets used in this study have been made freely available as part of their original publications. The DCIS data was obtained by Risom et al. (reference 15 in the manuscript) and is publicly available from Mendeley (DOI: 10.17632/d87vg86zd8.3). The CTCL data was obtained by Phillips et al.(reference 19 in the manuscript)

and is publicly available as Source data from the manuscript. The BC data was obtained from Jackson et al. (reference 30 in the manuscript) and is publicly available from Zenodo (DOI: 10.5281/zenodo.3518283). The PDAC data was obtained from Shiau et al. (reference 42 in the manuscript) and is publicly available from Mendeley (DOI: 10.17632/kx6b69n3cb.1). Source data are provided with this paper.

Research involving human participants, their data, or biological material

Policy information about studies with human participants or human data. See also policy information about sex, gender (identity/presentation), and sexual orientation and race, ethnicity and racism.

Reporting on sex and gender	n/a
Reporting on race, ethnicity, or other socially relevant groupings	n/a
Population characteristics	n/a
Recruitment	n/a
Ethics oversight	n/a

Note that full information on the approval of the study protocol must also be provided in the manuscript.

Field-specific reporting

Please select the one below that is the best fit for your research. If you are not sure, read the appropriate sections before making your selection.

[x] Life sciences [ ] Behavioural & social sciences [ ] Ecological, evolutionary & environmental sciences

For a reference copy of the document with all sections, see nature.com/documents/nr-reporting-summary-flat.pdf

Life sciences study design

All studies must disclose on these points even when the disclosure is negative.

Sample size	The DCIS data consisted of 79 samples coming from 70 patients. The CTCL data consisted of 69 samples coming from 14 patients. The Basel cohort of the BC data consisted of 376 samples coming from 28 patients. The PDAC data consisted of 300 fields of view (samples) coming from 13 patients.
Data exclusions	Samples were selected randomly matching two out of all possible categories per dataset while maximizing the number of samples and ensuring balance of categories for the analysis. From the DCIS data we selected 58 samples coming from 58 patients that were associated with either progressor (n = 14) or nonprogressor (n = 44) status. Samples from patients with normal and IBC-recurrence condition were excluded. From the CTCL data we selected 29 pre-treatment samples coming from 14 patients taken pretreatment and were associated with responder (n = 14) or nonresponder (n = 15) status. The post-treatment samples were excluded. From the Basel cohort of the BC data we selected 85 samples coming from the tumor region of 82 patients with ER+PR+HER2- clinical subtype, treated with hormonal therapy and associated with sensitive (n = 68) or resistant (n = 17) response. To obtain a balanced dataset we further randomly subselected 15 samples from each group. From the PDAC data we removed samples from one treated patient that in addition to the capecitabine or 5-fluorouracil treatment also received losartan treatment.
Replication	All random sample selection was seed controlled and is fully reproducible. The sample subselection and fold selection for cross-validation was performed with a single seed, making it deterministic and reproducible.
Randomization	The samples per dataset, where subselected, were selected randomly (seed controlled) from each group.
Blinding	Not applicable. All sample information was taken from the original studies.

Reporting for specific materials, systems and methods

We require information from authors about some types of materials, experimental systems and methods used in many studies. Here, indicate whether each material, system or method listed is relevant to your study. If you are not sure if a list item applies to your research, read the appropriate section before selecting a response.

Materials & experimental systems

n/a	Involved in the study
☒	☐ Antibodies
☒	☐ Eukaryotic cell lines
☒	☐ Palaeontology and archaeology
☒	☐ Animals and other organisms
☒	☐ Clinical data
☒	☐ Dual use research of concern
☒	☐ Plants

Methods

n/a	Involved in the study
☒	☐ ChIP-seq
☒	☐ Flow cytometry
☒	☐ MRI-based neuroimaging

Plants

Seed stocks

Report on the source of all seed stocks or other plant material used. If applicable, state the seed stock centre and catalogue number. If plant specimens were collected from the field, describe the collection location, date and sampling procedures.

Novel plant genotypes

Describe the methods by which all novel plant genotypes were produced. This includes those generated by transgenic approaches, gene editing, chemical/radiation-based mutagenesis and hybridization. For transgenic lines, describe the transformation method, the number of independent lines analyzed and the generation upon which experiments were performed. For gene-edited lines, describe the editor used, the endogenous sequence targeted for editing, the targeting guide RNA sequence (if applicable) and how the editor was applied.

Authentication

Describe any authentication procedures for each seed stock used or novel genotype generated. Describe any experiments used to assess the effect of a mutation and, where applicable, how potential secondary effects (e.g. second site T-DNA insertions, mosiacism, off-target gene editing) were examined.
